# Eyeing up the Future of the Pupillary Light Reflex in Neurodiagnostics

**DOI:** 10.3390/diagnostics8010019

**Published:** 2018-03-13

**Authors:** Charlotte A. Hall, Robert P. Chilcott

**Affiliations:** Research Centre for Topical Drug Delivery and Toxicology, University of Hertfordshire, Hatfield SP10 1JX, UK; c.hall5@herts.ac.uk

**Keywords:** pupillometry, acetylcholine, cholinergic system, neurodegeneration, trauma, infection, recreational drugs, chemicals, toxins, autism

## Abstract

The pupillary light reflex (PLR) describes the constriction and subsequent dilation of the pupil in response to light as a result of the antagonistic actions of the iris sphincter and dilator muscles. Since these muscles are innervated by the parasympathetic and sympathetic nervous systems, respectively, different parameters of the PLR can be used as indicators for either sympathetic or parasympathetic modulation. Thus, the PLR provides an important metric of autonomic nervous system function that has been exploited for a wide range of clinical applications. Measurement of the PLR using dynamic pupillometry is now an established quantitative, non-invasive tool in assessment of traumatic head injuries. This review examines the more recent application of dynamic pupillometry as a diagnostic tool for a wide range of clinical conditions, varying from neurodegenerative disease to exposure to toxic chemicals, as well as its potential in the non-invasive diagnosis of infectious disease.

## 1. Introduction

The origin of the phrase “the eyes are the window to the soul” is attributed to the Roman Consul Cicero, but during the past three decades the ability of the eye to act as a window into nervous system function has been exploited for a wide range of clinical applications, including mental health and neurodegenerative disorders, as well as exposure to toxic or illicit substances and trauma.

The pupillary light reflex (PLR) describes the constriction and subsequent dilation of the pupil in response to light, which not only serves as a major determination of retinal image quality [1,2], but also provides an important metric of autonomic nervous system function [3]. As such, measurement of the pupil’s response to light serves as a non-invasive tool for basic neuroscience research and the study of parasympathetic and sympathetic balance.

## 2. Pupillary Light Reflex

The pupil has a large dynamic range, typically from 7.5–8 mm diameter at full mydriasis to 1.5–2 mm diameter at full miosis, and is controlled by the antagonistic actions of the iris sphincter and dilator muscles [4]. The sphincter and dilator are innervated by the parasympathetic and sympathetic nervous systems, respectively; thus, different parameters of the PLR can be used as indicators for either sympathetic or parasympathetic modulation. Factors which affect average pupil diameter include age, sex, iris colour, retinal and optic nerve health and optical media clarity [5]; however, the most powerful determinant of pupil size is ambient light level. 

## 3. Measuring the PLR

**Figure 1:**
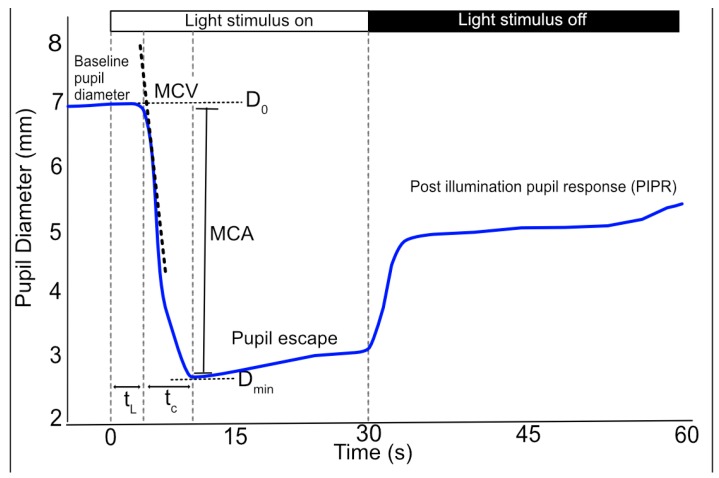
Schematic of the pupillogram (blue line) and associated PLR parameters. The light stimulus at time zero results in a rapid reduction in pupil diameter. Latency (t_L_) is calculated as the elapsed time between light onset and the start of constriction. The pupil then rapidly constricts (maximal constriction velocity; MCV) from the baseline (D_0_) pupil diameter to the minimum (D_min_) pupil diameter; the constriction time (t_C_) and maximum constriction amplitude (MCA) are calculated as the time interval and size difference between these two values, respectively. At offset of light stimulus or during sustained light stimulation the pupil undergoes a period of rapid redilation or pupillary “escape” to a partially constricted state. Subsequently the pupil slowly returns to the baseline diameter.

The dynamics of the PLR follow a general pattern consisting of 4 phases: response latency, maximum constriction, pupil escape and recovery (Figure 1), which can be influenced by the duration, intensity and spectral composition of the light. The PLR provides a physiological measure of normal or abnormal nervous system function and the symmetry of the PLR in response to stimulation of either eye, because of pupillary fibre decussation, provides an opportunity to compare the pupillomotor drive in both eyes [6].

Response latency describes the delay in pupil constriction following the onset of a light stimulus, with the latency shortening as light intensity increases, to a minimum of 180–230 ms [7,8]. The latency period is due to the delay in iris smooth muscle contraction and to a lesser extent the temporal dynamics of retinal output and innervation pathways [7,8]. 

The latency period is followed by a period of rapid constriction of the pupil until it reaches the maximum constriction velocity (MCV), after which constriction slows until the minimum pupil diameter is reached. The onset of pupil contraction can be determined using velocity and acceleration analysis [9]. The maximum constriction velocity varies with light stimulus intensity, duration, spectral composition, retinal size and location [10]. The maximum constriction amplitude (MCA) represents the difference between the baseline and minimum pupil diameter. However, the baseline pupil diameter can be affected by a number of factors and can influence the MCA (a smaller MCA is observed with a smaller baseline pupil diameter). Therefore, the MCA should be normalised to baseline pupil diameter to account for this effect [6]. After this peak constriction, the pupil quickly redilates or “escapes” to a partially constricted state during a prolonged light stimulus lasting from 1–2 up to 100 s, before slowly redilating to the initial size [11].

In addition to the dynamic phases of the PLR during light stimulation, there is also a sustained component [12,13]. Both outer and inner photoreceptors contribute to the early sustained post-illumination pupil response (PIPR; <1.7 s post-stimulus) [14]. Subsequently, the PIPR, which may be sustained for up to 3 minutes after light offset depending on the properties of the light stimulus, is solely controlled by the intrinsically photosensitive retinal ganglion cells that depolarise during light stimulation (dependent on both light intensity and wavelength) and then repolarise slowly after light offset [6,13,14,15]. 

The response of the pupil to light can be manually assessed using the swinging flashlight test or using a pupillometer device. Pupillometers enable objective quantification of the PLR and generally consist of an infrared-sensitive imaging sensor coupled with a digital interface for the automated recording, processing and reporting of pupil data. The acquired images are processed to yield a pupillogram with pupil size plotted as a function of time, from which PLR parameters can be calculated (Figure 1). There are several commercially available pupillometers and the relative performance of commercial systems is the subject of several publications [16,17,18]. However, commercial systems are typically designed for specific applications and use proprietary software, which may limit their use for basic research. In response, a number of research groups have published methods for developing prototype automated infrared pupillometers, which use open-source software [19,20]. There is also increasing use of remote eye-tracking devices, such as Tobii, that are capable of measuring pupil size and can be used in combination with open-source software for the analysis of PLR [21].

## 4. Neuronal Basis for the Pupillary Light Reflex

### 4.1. Pupil Constriction

There are three major divisions of parasympathetic neurons that integrate the light stimulus to produce a pupil contraction: (i) an afferent division; (ii) an interneuron division; and (iii) an efferent division. The response latency, maximum constriction and pupil escape, and the corresponding constriction parameters (MCV, MCA and RCA; relative constriction amplitude) are dependent on the actions of the sphincter muscle and on the function of retinal photoreceptors, as well as the time consumed in the afferent and efferent pathway. They are thus under direct control of the parasympathetic nervous system. Both MCV and RCA parameters are dependent on the baseline pupil diameter in healthy individuals and it is important to normalise measurements with respect to baseline values [6,22]. There is also a strong linear relationship between MCV and MCA [20]; thus, MCV and RCA are considered to be the most robust parameters for detecting parasympathetic dysfunction [23].

### 4.2. Afferent Arm of Pupil Constriction

**Figure 2:**
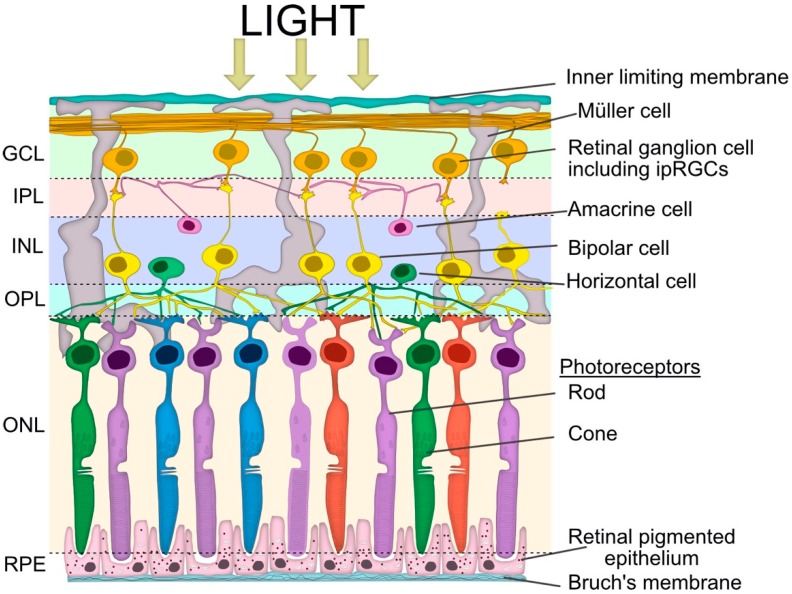
Simplified schematic view of retinal layers involved in the pupillary light reflex. Vertical signalling pathways in the retina are composed of the photoreceptors (rod and cone cells), bipolar cells and retinal ganglion cells (RGC), including intrinsically photosensitive retinal ganglion cells (ipRGCs). There are also two lateral pathways comprised of horizontal cells in the outer plexiform layer (OPL) and the amacrine cells in the inner plexiform layer (IPL). These cells modulate the activity of other retinal cells in the vertical pathway. The somata of the neurons are in three cellular layers. The rod and cone cells are located in the outer nuclear layer (ONL), which is adjacent to the retinal pigment epithelium (RPE). The horizontal cell, bipolar cell and amacrine cell somas are located in the inner nuclear layer (INL), whilst the ganglion cell somata are located in the ganglion cell layer (GCL). The axon terminals of the bipolar cells stratify at different depths of the inner plexiform layer, which is subdivided into the OFF outer sublamina (where OFF bipolar cells terminate) and the ON inner sublamina (where ON bipolar cells terminate). There are also ON and OFF bands of melanopsin dendrites from the ipRGCs, but both lie outside of the ON and OFF cholinergic bands within the IPL. The bipolar cells are photoreceptor specific and the bipolar dendrites synapse exclusively with either rod or cone cells.

The afferent limb of the PLR begins with photoreceptive inputs from rod, cone and intrinsically photosensitive retinal ganglion cells (ipRGCs) located in the retina (summarised in Figure 2).

Under dark conditions, rod and cone photoreceptors exist in a constant depolarised state and continuously release glutamate [24,25]. However, when stimulated by light, the rod and cone photoreceptors undergo graded alterations in membrane potential and hyperpolarize, resulting in a reduction in glutamate release. Rod and cone cells synapse with their respective bipolar cells and reductions in glutamate release results in either inhibition or disinhibition of the bipolar cells depending on whether they express excitatory ionotropic (“ON”) or inhibitory metabotropic (“OFF”) glutamate receptors [26]. These “ON” or “OFF” bipolar cell subtypes synapse with the corresponding retinal ganglion cell (RGC) in the non-cholinergic regions of the inner plexiform layer (IPL). Horizontal neurotransmission also occurs within the IPL and outer plexiform layer (OPL) via amacrine and horizontal cells, respectively, and is critical in shaping the spatial and temporal aspects of photopic and scotopic vision [27].

The third photoreceptor, ipRGC, accounts for approximately 1% of the total RGCs [28,29,30]. These cells have a role in non-image-forming visual processes, such as circadian photoentrainment, as well as in the PLR [31,32,33]. The ipRGCs regulate pupil size through the integration of extrinsic signals from rods and cones but also through intrinsic (melanopsin) phototransduction [12]. Melanopsin is a G protein-coupled photopigment, which is maximally sensitive to 482 nm wavelength light and, unlike rods and cones, depolarizes in response to light following activation of a phototransduction cascade involving Gq/11 and phospholipase C [34,35,36,37]. Unlike rods and cones, which have their photopigment concentrated in specialised light-absorbing cellular domains (outer segment), melanopsin is distributed throughout the plasma membrane of ipRGCs [28]. The ipRGCs also directly contribute to the PIPR as a sustained constriction of the PLR (>30 s) in response to high intensity, short wavelength light [14,15,28,38,39,40].

### 4.3. The Interneuron and Efferent Arms of Pupil Constriction

**Figure 3:**
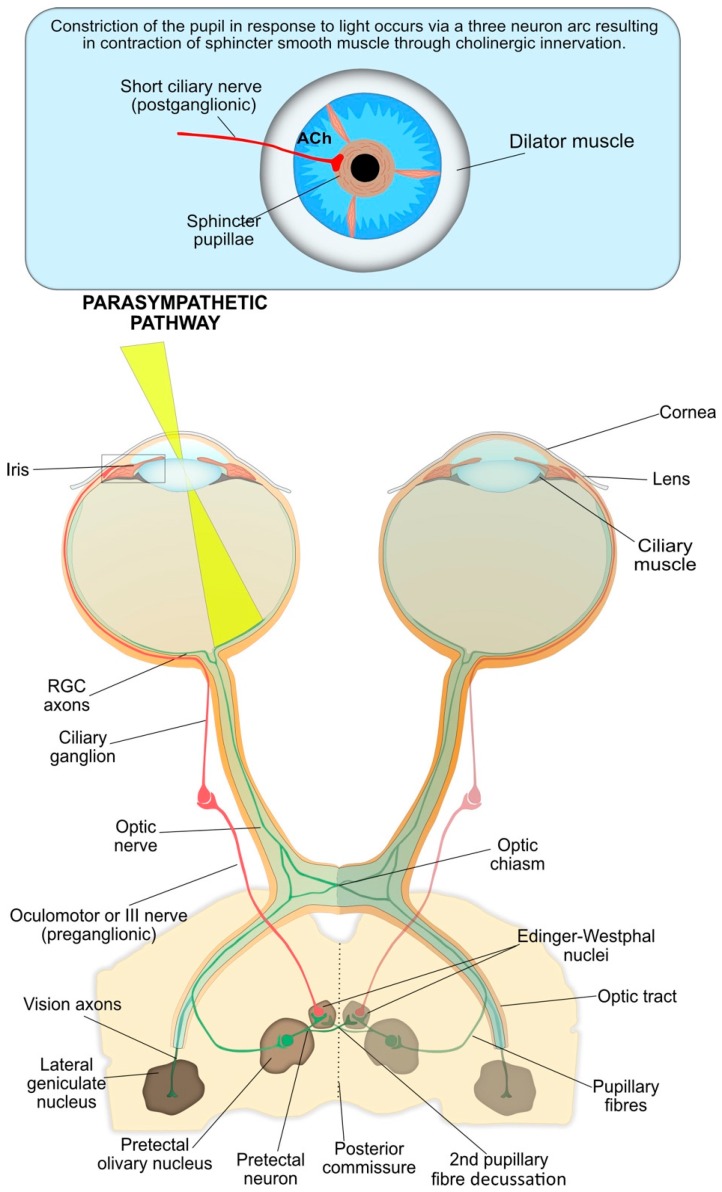
The parasympathetic nervous system is the main system responsible for pupil constriction in response to light. The integrated afferent input is transmitted along the axons of the retinal ganglion cells (RGC), which contribute to the optic nerve. At the optic chiasm, nerves from the nasal retina cross to the contralateral side, whilst nerves from the temporal retina continue ipsilaterally. The pupillary RGC axons exit the optic tract and synapse at the pretectal olivary nucleus. Pretectal neurons are projected either ipsilaterally or contralaterally, across the posterior commissure, to the Edinger-Westphal nucleus. From there, the pre-ganglionic parasympathetic fibres travel with the oculomotor, or III cranial nerve, and synapse at the ciliary ganglion. The post-ganglionic parasympathetic neurons (short ciliary nerves) travel to and innervate the contraction of the iris sphincter muscle via the release of acetylcholine at the neuromuscular junction, resulting in pupil constriction.

The interneuron and efferent arms of pupil constriction are summarised in Figure 3. The RGC axons form the first interneuron arm of the PLR arc and carry the neuronal signal from the photoreceptors [2].

At the optic chiasma, approximately half the RGCs from the nasal plane in each eye decussate to the opposite optic tract [41]. At the terminus of the optic tract, the axons of RGCs responsible for the PLR separate from the visual axons and carry the afferent pupillomotor signal through the brachium of the superior colliculus to synapse at the pretectal olivary nucleus in the dorsal midbrain [8].

The pretectal neurons integrate the input signals (retinal, supranuclear and infranuclear), that modulate the PLR and form the second interneuron of the reflex arc. These pretectal nuclei project to either the ipsilateral or contralateral Edinger–Westphal (EW) nucleus within the oculomotor nuclear complex, which contain the pre-ganglionic parasympathetic neurons that control the iris sphincter [42]. The bilateral neuron projection results in a double decussation of pupillary fibres, first at the optic chiasm and then within the pretectal area, and ensures each EW nucleus receives information about the level of incoming light from each eye. Therefore, unilateral light stimulation causes an equal direct and consensual pupillary constriction. However, contraction anisocoria, whereby the direct pupillary constriction is slightly stronger than the consensual reaction, may present if asymmetry occurs during crossing of fibres at the chiasm or pretectal olivary nucleus; this is normally clinically insignificant [8].

From the EW nuclei, the efferent pre-ganglionic axons pass into the right and left fascicles of the oculomotor nerve (third nerve) to join the motor axons destined for the eye muscles (Figure 3). The oculomotor nerve bifurcates into a superior and inferior division near the anterior cavernous sinus. The parasympathetic fibres travel with the inferior division through the superior orbital fissure toward the orbital apex and synapse at the ciliary ganglion (CG).

The short ciliary (post-ganglionic) nerves pierce the globe around the optic nerve and pass between the choroid and sclera toward the iris. These nerves innervate the contraction of the iris sphincter muscle via the neurotransmitter acetylcholine (ACh), resulting in constriction of the pupil.

### 4.4. Pupil Reflex Dilation: Central and Peripheral Nervous System Integration

**Figure 4:**
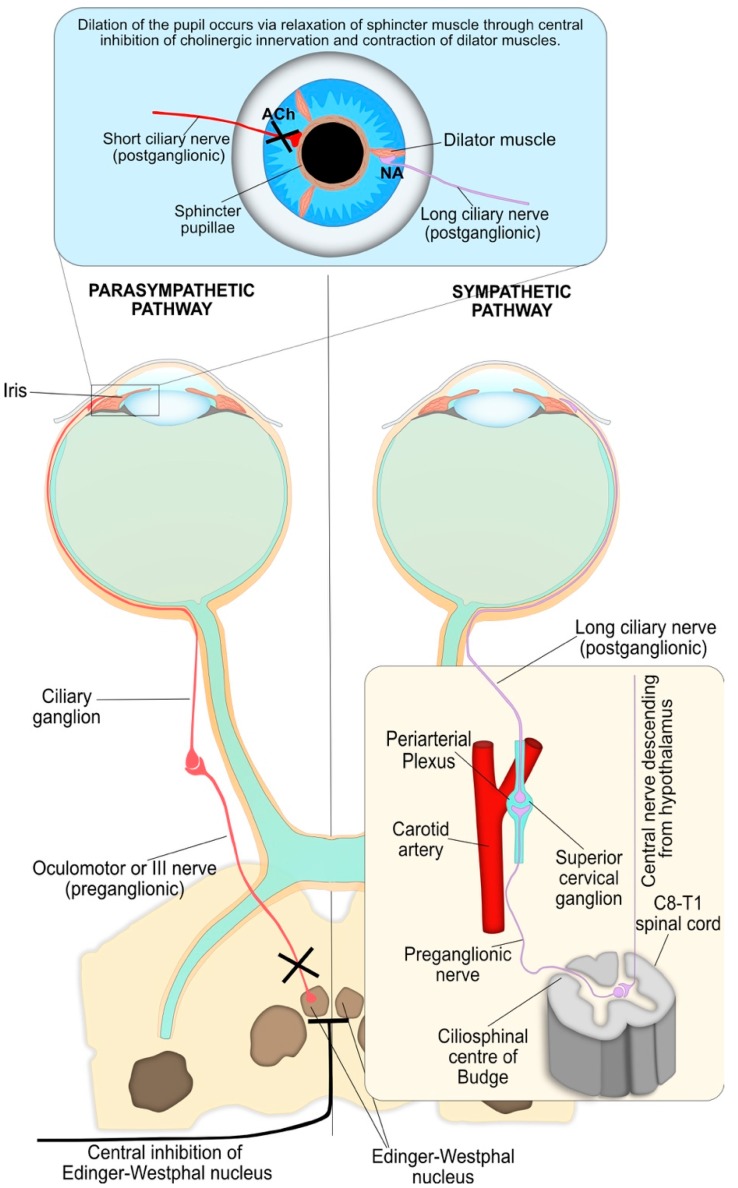
Both parasympathetic and sympathetic nervous systems are required for pupil dilation as part of the PLR. The parasympathetic innervation of the pupil sphincter is inhibited by central supranuclear inhibition of Edinger–Westphal nuclei via α_2_-adrenergic receptor activation, resulting in relaxation of the pupil sphincter muscle. The sympathetic influence on the iris dilator muscle consists of a paired three-neuron arc on both the right and left sides of the central and peripheral nervous system without decussations. The first-order (central) neuron originates in the hypothalamus and descends to synapse with the pre-ganglionic in the ciliospinal centre of Budge at C8-T1 of the spinal cord. The pre-ganglionic neuron ascends from the ciliospinal centre of Budge to synapse with the post-ganglionic neuron at the superior cervical ganglion, which is located at the periarterial plexus near the carotid artery bifurcation. Finally, long ciliary (post-ganglionic) nerves travel to and innervate the contraction of the iris dilator muscles, via a release of noradrenaline (NA) at the neuromuscular junction, resulting in pupil dilation. The synaptic transmission at the other junctions is mediated by acetylcholine.

Dilation of the pupil following a light stimulus occurs through two integrated processes driven by the sympathetic neurons and corresponds to the recovery phase of the PLR and is summarised in Figure 4. Firstly, the parasympathetic innervation of the pupil sphincter is suppressed by supranuclear inhibition via central sympathetic neurons, resulting in relaxation of the muscle and pupil dilation. These sympathetic neurons primarily originate in the reticular activating formation in the brainstem and inhibit the pre-ganglionic parasympathetic neurons at the EW nucleus via α_2_-adrenergic receptor activation. Secondly, the iris dilator muscle contracts via excitation of the α_1_-adrenergic sympathetic pathway. This peripheral sympathetic nerve activation greatly enhances the dynamics of pupil dilation in terms of speed and maximal pupil diameter attained.

The sympathetic influence on the iris dilator muscle consists of a paired, three-neuron arc on both the right and left side of the central and peripheral nervous system, without decussations, extending from the hypothalamus to the iris dilator muscle (Figure 4) [43,44,45]. In this three-neuron arc, the synaptic transmission is mediated by ACh at the first two junctions, while the post-ganglionic fibres innervate the dilator muscle via noradrenaline. 

### 4.5. Other Inputs to the Iris

Nerves within the ophthalmic division of the trigeminal nerve provide sensory innervation to the iris and may play an additional role in modulating pupil diameter [46]. Mechanical and chemical irritation of the eye can cause a strong miotic response that is non-cholinergic and fails to reverse with autonomic-acting drugs. In addition to the neuronal mechanisms involved in pupil size control, circulating catecholamines and peptide hormones may act on the iris dilator or sphincter muscles, either directly through the bloodstream or potentially indirectly through the tears [47,48].

## 5. Clinical Applications of Pupillometry

Conditions that influence the integration of parasympathetic stimulation and inhibition, sympathetic stimulation and humoural release of neurotransmitters may each affect the dynamics of the PLR and may be clinically diagnostic. Pupil function abnormalities have been reported for a wide range of disorders, including alcoholism [49,50], mental health disorders such as seasonal affective disorders [51], schizophrenia [52] and generalised anxiety disorder [53], Alzheimer’s [54,55,56] and Parkinson’s [57,58,59,60] diseases, autism spectrum disorders [61,62], as well as glaucoma [14,63,64,65] and autonomic neuropathies associated with diabetes [66,67,68,69,70]. Additionally, pupillometry has been applied to other clinical fields, such as monitoring of central states in anaesthesiology and analgesia, as well as monitoring and prognosis following head injuries, cardiac arrest and drug overdose [71].

### 5.1. Neurodegenerative Disorders

There is an established body of evidence to indicate that cholinergic hypofunction is a significant component of neurodegenerative diseases, such as Alzheimer’s and Parkinson’s diseases, which are due to ACh and dopamine deficiencies, respectively. Moreover, the majority of studies that analysed ACh-dependent PLR parameters have revealed significant differences between normal age-matched patients and those with Alzheimer’s [55,56,57,72,73,74] or Parkinson’s disease [58,59,75]. Studies typically used light stimuli wavelength of 820 nm with a light intensity of 24.6 cd/m^2^. Specifically, patients with Alzheimer’s [57] or Parkinson’s disease, with or without any detectable cognitive deficits or psychiatric disorders [55,57,75], had significantly lower MCV and MCA values. Ferrario et al. [76] did not observe a significant difference in MCA between Alzheimer’s patients and controls; however, this may be attributed to the longer light stimulus used (1 s) compared to the other studies (20–150 ms) [77].

The MCV and MCA parameters correspond to the first portion of the characteristic V-shaped pupillometric response (Figure 4) and are considered the most sensitive markers of cholinergic activity [78]. Therefore, the MCA and secondarily the MCV are the best PLR predictors to discriminate between healthy individuals and patients with Alzheimer’s or Parkinson’s disease [57,72]. A significant increase in latency has been observed for subjects with Parkinson’s disease [57,58,59]; however, this was not observed consistently in those with Alzheimer’s disease [54,56,72]. Additionally, Bittner et al. examined the pupil’s response under repetitive light stimulation, which is systematically unstable in normal patients [54]. However, these changes were not observed in patients with Alzheimer’s disease.

A number of possible pathophysiological mechanisms have been proposed for the observed changes in pupillary response seen in Alzheimer’s and Parkinson’s patients [60], but Fotiou et al. [57] and others have concluded that the most significant factor behind these findings was likely to be the involvement of a central cholinergic deficit. Pupillometry, including repeated light stimulation pupillometry, may serve as a useful diagnostic tool even at early, subclinical stages of autonomic nervous system dysfunction [57,58].

### 5.2. Trauma

The PLR is a well-established measurement in the management and prognosis of patients with acute brain injuries, in conjunction with other clinical parameters such as age, mode of injury and Glasgow Coma Scale [79,80,81]. Typical light stimuli parameters are a 465 nm wavelength with a duration of 1 s and low luminance (0.001 cd/m^2^) used to stimulate rod cells or high luminance (450 cd/m^2^) used to stimulate ipRGCs. In particular, the location of the pupillomotor nuclei within the dorsal midbrain and efferent oculomotor nerve are important in the determination of brainstem compression and the onset of transtentorial herniation [82]. Morris et al. reported that loss of the PLR or development of anisocoria or pupil asymmetry >2 mm in patients who sustained traumatic brain injuries was correlated with increased morbidity and mortality rates [83]. However, manual examination using a penlight is subject to large inter-examiner discrepancies that can be as high as 40%, particularly when pupils are constricted [84], and these may be further confounded by a variety of factors, including alcohol, narcotics or hypothermia, which are common to many trauma patients [85]. Furthermore, Couret et al. observed an error rate of approximately 20% even for intermediate-sized pupils (2–4 mm), with a 50% failure rate in the detection of anisocoria [86]. Additionally, Larson and Muhiudeen found complete failure in the detection of the PLR by manual examination when the reflex amplitude was less than 0.3 mm [87]. Automated pupillometry using a pupillometer is a more sensitive technique that has smaller inter-examiner discrepancies compared to manual examination [86,88]. The ability of pupillometry to detect subtle changes in pupillary reaction even when pupils are constricted has potential clinical significance and may provide a useful tool in the early detection, monitoring and management of brain injuries [89]. In support of this, several groups have demonstrated that use of a pupillometer was superior to manual assessment in predicting the 90-day outcome following cardiac arrest [90,91].

### 5.3. Autism

The cholinergic system is key to normal pre- and postnatal neurodevelopment, and numerous studies, ranging from neuroimaging data [92], to post mortem histopathological analysis of brain tissue [93,94], animal models [95,96] and molecular genetic studies [97], have suggested that alterations in the cholinergic system may be a contributing factor to the aetiology of autism spectrum disorder (ASD). Moreover, an atypical PLR is reported in both children and adults with ASD [60,98,99,100].

Common measurement parameters used include a light stimulus wavelength of 530 nm and a duration of 100 ms with a light intensity of 63.1 cd/m^2^. Typically, these differences are characterised by longer latency, reduced constriction amplitude [61,99] and reduced constriction velocity [61] compared to children without ASD. However, Nyström et al. reported the opposite for high ASD-risk infants, defined as those who had a sibling with ASD. The difference in age among the studies’ subjects, who ranged from 10-month-old infants [100] up to children >5 years of age [61,98,99], may provide an explanation for the contrasting data.

Children without ASD exhibit an age-dependent decrease in PLR latency before reaching a plateau at >8 years of age [98,99]; this correlates with similar trends in white-matter maturation rates as determined by flash visual evoked potential studies [101]. However, in children with ASD there was no age-dependent decrease in PLR latency [99]. Moreover, brain-imaging studies have shown that the neurodevelopment trajectory of brain maturation is atypical in children with ASD. Initially, young children (<4 years of age) exhibit accelerated maturation and a larger brain volume compared to children without ASD [102,103,104,105,106], but this is followed by a period of arrested maturation after 4 years of age [104,107], and then a possible decrease in brain volume in older children and adults [108]. Therefore, the hypersensitive PLR observed in infants with a high risk of ASD [100] could be attributed to the accelerated white-matter maturation associated with ASD, which then reverses after 4 years of age, resulting in a hyposensitive PLR response corresponding to the reduction in PLR parameters [61,98,99].

There is evidence to suggest that, apart from the cholinergic system, other neurotransmitter systems are altered in ASD, such as glutamatergic and GABAergic transmission [109]. Therefore, the PLR may also be affected as a result of altered bipolar cell signalling within the retina. Overall, assessment of the PLR by pupillometry may provide a rapid and non-invasive diagnostic tool for infants and children with a high risk of ASD or other cholinergic-dependent neurological development disorders [100].

### 5.4. Alcohol and Recreational Drugs

Pupillometry offers a reliable and convenient tool for illicit drug and alcohol screening [110,111,112]. A number of studies have demonstrated the ability of pupillometry to differentiate between potentially drug impaired and normal subjects with 70–100% accuracy [113,114]. The most significant parameters were RPA and MCV [112]. Additionally, pupillometry may offer benefits over urinalysis, particularly in the case of roadside testing, as it provides a measurement of function and impairment rather than a measurement of drug metabolites, which may be present in the urine for an extended period even though any drug-induced physiological effects or impairment have ceased.

#### 5.4.1. Alcohol

Pupillometry may have a role in both the detection of alcohol intoxication and treatment management during alcohol withdrawal. Data from chromatic pupillometry studies demonstrated a significant increase in both baseline pupil diameter and peak constriction amplitude following a 600 nm wavelength light stimulus at exhaled breath alcohol concentrations of ≥0.25 mg/L [115]. However, following a high dose of alcohol (1 g/kg body weight) the opposite was observed, with significant decreases in pupil diameter, constriction amplitude and velocity compared to control groups, suggesting inhibition of parasympathetic nerve activity [116,117]. These apparently contradictory results are likely to reflect the acute, dose-dependent inhibition of the parasympathetic nervous system, which results in the predominance of sympathetic nerve activity [118].

Pupillometry may also have a role in the development of clinical management tools to prevent severe autonomic dysfunction during alcohol withdrawal [119]. Specifically, prolonged latency and decreased constriction velocity parameters were described for participants undergoing alcohol withdrawal. The reduction in parasympathetic innervation of the pupil is likely to be due to increased activation of the locus coeruleus, as previously described during alcohol withdrawal [120,121].

#### 5.4.2. Recreational Drugs

The pupillary response to 3,4 methylenedioxymethamphetamine (MDMA) and tetrahydrocannabinol (cannabis) is characterised by an indirect central parasympathetic inhibition, resulting in significantly increased latency and decreased constriction amplitude and velocity [113,122,123]. Additionally, increased sympathomimetic activity due to increased noradrenaline and serotonin signalling was reported following MDMA intoxication, resulting in mydriasis and a reduction in the PLR recovery time [123].

However, for cannabis intoxication there are conflicting reports regarding the effect of the drug on baseline pupil diameter. Hartman et al. observed a significantly increased pupil size compared to control participants, under both scotopic and photopic light conditions as well as following direct light stimulation, suggesting increased sympathetic nervous system activity [112]. This is in contrast to data reported by Fant et al. and others, which demonstrated a significant cannabis-induced effect on the PLR but either a small (0.5 mm) decrease [116,124] or no change [122] in baseline pupil diameter. These differences may be attributed to study design, as the studies that observed little or no effect on pupil diameter used a defined, high dose (27 mg Δ^9^THC) and a specified time duration between drug administration and pupil measurements, whereas Hartman et al. used Drug Recognition Expert examination data; thus, the exact dose and timings are undefined [113,116,122].

### 5.5. Exposure to Toxins and Toxic Chemicals

Changes in pupil size and response to light have been reported following exposure to toxic chemicals such as organophosphates, as well as bacterial toxins including botulinum toxin.

Ophthalmic manifestations are early and persistent signs of botulism. Botulinum toxins (BTx) block the release of ACh at neuromuscular junctions, post-ganglionic parasympathetic nerve endings, and post-ganglionic sympathetic nerve endings that release Ach, resulting in paralysis of the sympathetic and parasympathetic innervation of the iris [125,126]. This may result in transient pupil dilation and attenuation of the PLR by uptake of BTx into the parasympathetic ciliary ganglion or the parasympathetic neuromuscular junctions at the iris sphincter muscle [127]. There are a number of reports in the literature describing mydriasis with an attenuated PLR as a consequence of ingesting contaminated food [128], or following injection of BTx [129,130].

Organophosphates, a family of chemicals that includes nerve agents and pesticides, inhibit cholinesterase activity, resulting in increased levels of ACh at the nerve synapses; they thus act as an indirect cholinergic agonist. Published studies by Dabisch et al. and others suggest that the majority of cholinesterase inhibition observed within the eye is a result of the nerve agent vapour acting directly on the ocular tissues, rather than distributing to the eye as a result of systemic absorption [131,132,133]. The localised increase in ACh leads to contraction of the pupillary sphincter muscle, resulting in dose-dependent miosis [132,134,135,136]. Miosis is a highly sensitive index of exposure and can occur at exposure levels below those that cause systemic effects [137,138]. In relevant animal models, the amounts of sarin and cyclosarin required to produce miosis were up to 30- and 135-fold lower, respectively, than the amounts required for lethality [139].

The PLR is also reduced following organophosphate exposure, as a result of developing tolerance to cholinergic agonists and desensitization of muscarinic ACh receptors within retinal tissue following prolonged exposure [135,140,141]. The threshold dose required to attenuate the PLR is similar to that required to produce miosis, but the duration of the response is very different [140].

Dabisch et al. [140] observed rapid miosis and attenuation of the PLR in a rodent model following a single low-dose soman vapour exposure, but while pupil size returned to normal after 48 h, the PLR took up to 10 days to fully recover. Similarly, exposure to dichlorvos vapour resulted in a dose-dependent transient miotic response in the guinea pig; however, a persistent enhanced pupillary response to light was observed [136]. The recovery of pupil size is attributed to desensitisation of the muscarinic receptors rather than reactivation of cholinesterases within the eye, which may take up to 6 days to recover. Consequently, the PLR is attenuated until muscarinic receptor function is regained [142]. In support of this, oximes, which reactivate acetylcholinesterase, had no effect on sarin-induced miosis in animal models. Moreover, tropicamide—a muscarinic receptor antagonist that competes with ACh for binding sites, preventing receptor desensitisation—rapidly increased pupil size and restored PLR [143]. However, organophosphates inactivate cholinesterases at both muscarinic and nicotinic receptor sites and paradoxical pupil dilation or mydriasis may occur in certain circumstances due to dominant nicotinic effects at the pre-ganglionic fibres of the sympathetic nervous system, resulting in increased innervation of the dilator muscle [144,145].

### 5.6. Response to Infection

An area of interest that currently remains unexplored is the response of the PLR to infection and the potential diagnostic value of pupillometry. The brain monitors and modulates immune status through both humoural and neural pathways [146,147]. Neuroendocrine responses control inflammation at the systemic level through the hypothalamic-pituitary-adrenal axis [148]. The first branch of this pathway, the vagus nerve, is activated either directly by cytokines (released from innate immune cells) or indirectly through the chemoreceptive cells located in the vagal paraganglia [149]. The release of pro-inflammatory cytokines needs to be carefully controlled, as excessive or uncontrolled release (also known as a “cytokine storm”) may contribute to the pathogenesis of infections, including the novel coronaviruses SARS and MERS [150,151,152], influenza [153], and Ebola [154], as well as potential bacterial biothreat agents such as *Burkholderia pseudomallei* [155] and *Yersinia pestis* [156].

Signals from the vagus afferent fibres eventually project to the *locus coeruleus* region of the brain. The *locus coeruleus* exerts a dual influence on the PLR, ultimately leading to pupil dilation [157,158]. Firstly, it contributes to the sympathetic outflow that innervates the pupillary dilator muscle. Secondly, it attenuates the parasympathetic outflow via inhibition of the EW nucleus. Furthermore, experimental models of infection—including sepsis [159,160,161], pneumococcal pneumonia [162], endotoxaemia [163,164], leptospirosis [165,166] and influenza A [167,168]—have also highlighted the significance of the cholinergic signalling pathway during infection. Changes to cholinergic signalling are likely to influence the PLR both directly, through ACh receptors located on iris sphincter muscles, and indirectly, through altered parasympathetic nervous system function. Therefore, measurement of the PLR using dynamic pupillometry may offer the potential to detect systemic changes in parasympathetic and sympathetic system function in response to infection and inflammation.

## 6. Limitations

Pupillometry shows promise as a non-invasive diagnostic technology for a wide range of conditions. However, there are a number of limitations that require consideration and further research is needed to enable translation into clinical settings. Firstly, PLR measurements can be influenced by the light stimuli used [6,169], sex [170], age [171,172] and iris colour [173]. Changes in pupil size are also observed in response to other stimuli, including spatial structure patterns [174,175], object nearness or accommodation reflex [10], and a variety of emotional and cognitive stressors [176,177]. Therefore, it is critical that standardised protocols be developed to enable the use of pupillometry as a diagnostic tool and limiting factors should be considered as covariates or exclusion criteria in PLR studies to enable inter-study comparisons [23].

Further research is also required to establish whether the observed changes in PLR associated with different disorders are sufficient in terms of specificity and sensitivity to be used diagnostically. However, the ease of use, non-invasive nature and low cost mean pupillometry is well-placed for inclusion in early diagnostic screening assessments and could complement other sources of information to identify individuals at risk who warrant further clinic investigations. 

Furthermore, there are approaches that have been exploited in particular fields—such as the use of chromatic pupillometry to measure the response of different sub-types of photoreceptor, specifically ipRGCs, in diabetes [66] and glaucoma [14,64,65]—which could be applied to other conditions including neurodegenerative disorders. 

## 7. Conclusions

The pupillary light reflex serves as a valuable indicator of autonomic nervous system function. Moreover, measurement of the reflex using dynamic pupillometry provides a quantitative, non-invasive tool, which may aid the diagnosis and clinical management of a wide range of clinical conditions, varying from neurodegenerative disease to exposure to toxic chemicals.
